# Association of urinary albumin excretion with all-cause and cardiovascular mortality among patients with rheumatoid arthritis: a national prospective study

**DOI:** 10.3389/fimmu.2024.1412636

**Published:** 2024-09-19

**Authors:** Zexuan Bin, Ruihua Shen, Ruihe Wu, Yuxin Fan, Xin Zhang, Chong Gao, Xiaofeng Li, Caihong Wang

**Affiliations:** ^1^ Department of Rheumatology, The Second Hospital of Shanxi Medical University, Taiyuan, Shanxi, China; ^2^ Shanxi Key Laboratory of Immunomicroecology, Taiyuan, Shanxi, China; ^3^ Department of Nephrology, The Second Hospital of Shanxi Medical University, Taiyuan, Shanxi, China; ^4^ Pathology, Joint Program in Transfusion Medicine, Brigham and Women’s Hospital/Children’s Hospital, Harvard Medical School, Boston, MA, United States

**Keywords:** urine albumin to creatinine ratio, urinary albumin excretion, rheumatoid arthritis, mortality, cardiovascular disease

## Abstract

**Background:**

Rheumatoid arthritis (RA) patients suffering from chronic renal insufficiency tend to exhibit subtle manifestations at the beginning. Urine albumin to creatinine ratio (ACR) is a sensitive indicator for early assessment of renal function. However, it is unclear whether it serves as an independent risk factor influencing the prognosis of RA patients.

**Methods:**

National Health and Nutrition Examination Survey (NHANES) data from 2009-2018 were included. Kaplan-Meier (K-M) curves were plotted to compare the cumulative survival probability of RA patients with different urinary albumin excretion. The association of ACR with mortality among RA patients was investigated with Cox regression model, restricted cubic spline (RCS) and stratified analyses. The prognostic efficacy of ACR and estimated glomerular filtration rate (eGFR) was evaluated by receiver operating characteristic (ROC) curves.

**Results:**

The Cox regression model adjusted with covariates showed a 53% (HR 1.53, 95% CI 1.06-2.21) increase in all-cause mortality and a statistically non-significant increase in cardiovascular disease (CVD) mortality in RA patients with microalbuminuria (30mg/g ≤ACR<300mg/g). ACR≥300mg/g was associated with an increase in all-cause mortality (HR 2.62, 95% CI 1.55-4.45) and CVD mortality (HR 5.67, 95% CI 1.96-16.39). RCS demonstrated a nonlinear correlation between ACR and all-cause mortality in RA patients with microalbuminuria. Subgroup analysis showed that CVD mortality was higher in RA patients with microalbuminuria characterized by the following features: female, other ethnicity, eGFR≥60 ml/min/1.73 m2, hypertension or hyperlipidemia. Compared with eGFR, ACR provided better prognostic efficacy than eGFR with higher values of the area under the curve (AUC) for all-cause mortality (AUC=0.683, 95% CI 0.613-0.754) and CVD mortality (AUC=0.681, 95% CI 0.541-0.820).

**Conclusion:**

ACR is an independent risk factor affecting the prognosis of RA patients. The all-cause mortality was increased in RA patients with albuminuria. There was an upward trend in the CVD mortality of those with macroalbuminuria when ACR increased.

## 1 Introduction

Rheumatoid arthritis (RA) is a chronic autoimmune disease characterized by symmetrical small joint inflammation involving multiple organs throughout the body. Extra-articular manifestations occur during the progression of rheumatoid arthritis with multiple organs involved including heart, lungs, eyes, kidneys and so on, which seriously affect the prognosis of RA patients. Comorbid renal insufficiency in RA patients tends to be a persistent and sneaky condition. A cross-sectional multicenter study suggested 9% of 931 patients with RA had proteinuria and 8.8% had an eGFR <60 ml/min/1.73 m2 (1). However, when raised blood creatinine or large amounts of albuminuria are noticed, RA individuals usually have experienced kidney disease progression, which deteriorates their living conditions and brings therapeutic challenges. Renal insufficiency in patients with RA may be attributed to a variety of causes. As is known, some disease-modifying antirheumatic drugs (DMARDs) such as cyclosporine and methotrexate, are nephrotoxic. Besides, vasculitis and secondary renal amyloidosis degeneration after RA may trigger kidney damage such as concomitant nephrotic syndrome. Participants with chronic kidney disease (CKD) exhibit an alarming incidence of cardiovascular disease (CVD) events, especially adverse outcomes such as heart failure and malignant arrhythmias (2, 3). Compared with healthy controls, individuals with RA are associated with higher risk of cardiovascular events due to their highly inflammatory environments (HR 1.33, 95% CI 1.07 to 1.65, p=0.010) (4). Thus, it is essential for prediction of RA prognosis to early assess the risk of all-cause and CVD mortality associated with renal insufficiency.

The urine albumin to creatinine ratio (ACR) is a key indicator for albuminuria in CKD (5). The Kidney Disease Outcomes Quality Initiative (KDOQI) 2021 defines normal values for ACR as less than <30 mg/g, with 30-300 mg/g presenting as microalbuminuria, and those ≥300 mg/g as macroalbuminuria (6). ACR exceeding 30 mg/g and lasting for a period of greater than or equal to 3 months is considered to have CKD and a higher ACR is a significant marker of renal injury. Besides, a small increase above the normal range of ACR (30-300 mg/g) reflects the level of urinary microalbumin, which tends to indicate vascular damage and be strongly associated with cardiovascular complications in a variety of diseases (7–9). Several studies have suggested that in a normal population ACR was linearly correlated with the risk of mortality (10, 11). However, there is a lack of evidence on the relationship between ACR and mortality in RA cohorts. Besides, the relationship between adverse CVD occurrence and urinary albumin excretion in RA still requires further investigation. Other risk factors affecting RA such as demographics, lifestyle habits, and comorbidities should also be taken into account in order to demonstrate independent impact of ACR on mortality. Elucidating the association of ACR with all-cause and cardiovascular mortality among RA participants facilitates the investigation of novel, sensitive markers that predict poor prognosis and the evaluation of the effect of urine albumin excretion on RA prognosis.

## 2 Materials and methods

### 2.1 Study participants

National Health and Nutrition Examination Survey (NHANES) is a survey based on the health and nutritional status of adults and children in the USA. By demonstrating questionnaires, laboratory data, and health examinations, this platform provide a multidimensional landscape of USA population health conditions.

We combined the NHANES data from 2009-2018 for a total of 5 cycles, excluding respondents younger than 20 years of age, and excluded subjects with undocumented arthritis and ACRs from the 27,070 participants. 1,363 patients with RA were selected based on participants answered as “Rheumatoid arthritis” to the MCQ191 and MCQ195 question “What type of arthritis do you have”. 4 patients with missing mortality data and 77 patients without covariate data were excluded, and finally 1282 RA patients were enrolled in our study.

### 2.2 Albumin to creatinine ratio measurement

According to the NHANES website, urinary albumin and creatinine were measured by solid-phase fluorescence immunoassay and modified Jaffe kinetics. The ACR was calculated by dividing the urinary albumin concentration by the urinary creatinine concentration in mg/g. We selected the variable URDACT in the ALB_CR dataset of the laboratory data as the value of the ACR and classified the ACR into three intervals according to KDOQI guidelines for statistical analysis: <30 mg/g, 30-300 mg/g, and ≥300 mg/g, using the RA population with ACR <30 mg/g as a reference. ACR was tested at baseline in patients with RA.

### 2.3 Mortality

We collected information on mortality status and follow-up time (as of 31 December, 2019) through the National Death Index Mortality Database of NHANES. The primary outcomes in our study were all-cause mortality and cardiovascular mortality, with Causes of death determined according to the International Classification of Diseases, Tenth Revision (The codes for heart disease: I00–I09, I11, I13, and I20–I51).

### 2.4 Covariates

Covariates such as demographics, marital status, smoking status, education, diabetes, hypertension, hyperlipidemia and estimated glomerular filtration rate (eGFR) were obtained from NHANES questionnaire and laboratory data and included in the study. The poor population was defined as poverty to income ratio (PIR) <1. The married population was defined as being married or living with partner. Smokers were classified as smoking now or having smoking history. Diabetes was defined as taking hypoglycemic medication, using insulin, having been informed by a physician of a diagnosis of diabetes mellitus, having a hemoglobin A1c (HbA1c) level of ≥ 6.5%, or having a fasting glucose of ≥ 126 mg/dl. Hypertension was defined as being on antihypertensive medication, having ever been informed by a physician of a diagnosis of hypertension, having had three consecutive systolic blood pressure measurements of ≥ 140 mmHg, or diastolic blood pressure of ≥ 90 mmHg. Hyperlipidemia was defined as having total cholesterol >200 mg/dl, triglycerides >150 mg/dl, LDL >=130 mg/dl, HDL for men <40 mg/dL, <50 mg/dL for women, or subjects who had been informed by a physician of a diagnosis of hyperlipidemia. The eGFR was determined by the CKD Epidemiology Collaboration equation (12).

### 2.5 Data analysis

Data were analyzed by dividing RA patients into three groups according to ACR: <30 mg/g, 30-300 mg/g and ≥ 300 mg/g. General characteristics of the population were described by a baseline table. Differences between the three groups were compared using the Kruskal-Wallis test (non-normally distributed continuous variables), the χ^2^ test (categorical variables), and the Fisher’s exact probability test (counting variables with theoretical numbers <10). ACR is non-normally distributed. Kaplan-Meier (K-M) curves were plotted to visualize the survival status of RA patients. Hazard ratios (HR) values and 95% confidence intervals (95% CI) for RA patients were calculated with the Cox proportional risk model, and three models were developed: model 1 was unadjusted, model 2 was adjusted for age, sex, and race, and model 3 was adjusted for age, gender, race, marital status, poverty-to-income ratio (PIR), smoking, comorbidities and eGFR. The nonlinear relationship between urinary albumin excretion rate and mortality in RA patients was investigated by the restricted cubic spline (RCS) model for RA populations with ACR less than 300 mg/g. Models fitted by RCS were similarly adjusted for covariates as model 3. The RCS model revealed no correlation between ACR <300 mg/g and cardiovascular mortality in RA patients with microalbuminuria in the adjusted model (*P >*0.05). Further subgroup analyses of RA patients with ACR lower than 300 mg/g were carried out to investigate the effect of ACR on mortality. The effect of ACR on mortality among RA patients of different categories was studied based on populations stratified by demographic characteristics and comorbidities. Interactivity test and sensitivity analysis were used to assess model robustness. Finally, ROC curves revealed prognostic efficacy of ACR and eGFR for all-cause mortality and CVD mortality.

All statistical analyses in this study were performed using R (version 4.3.1). A two-tailed *P* value less than 0.05 is considered to be statistically significant.

## 3 Results

### 3.1 Baseline characteristics

**Figure 1:**
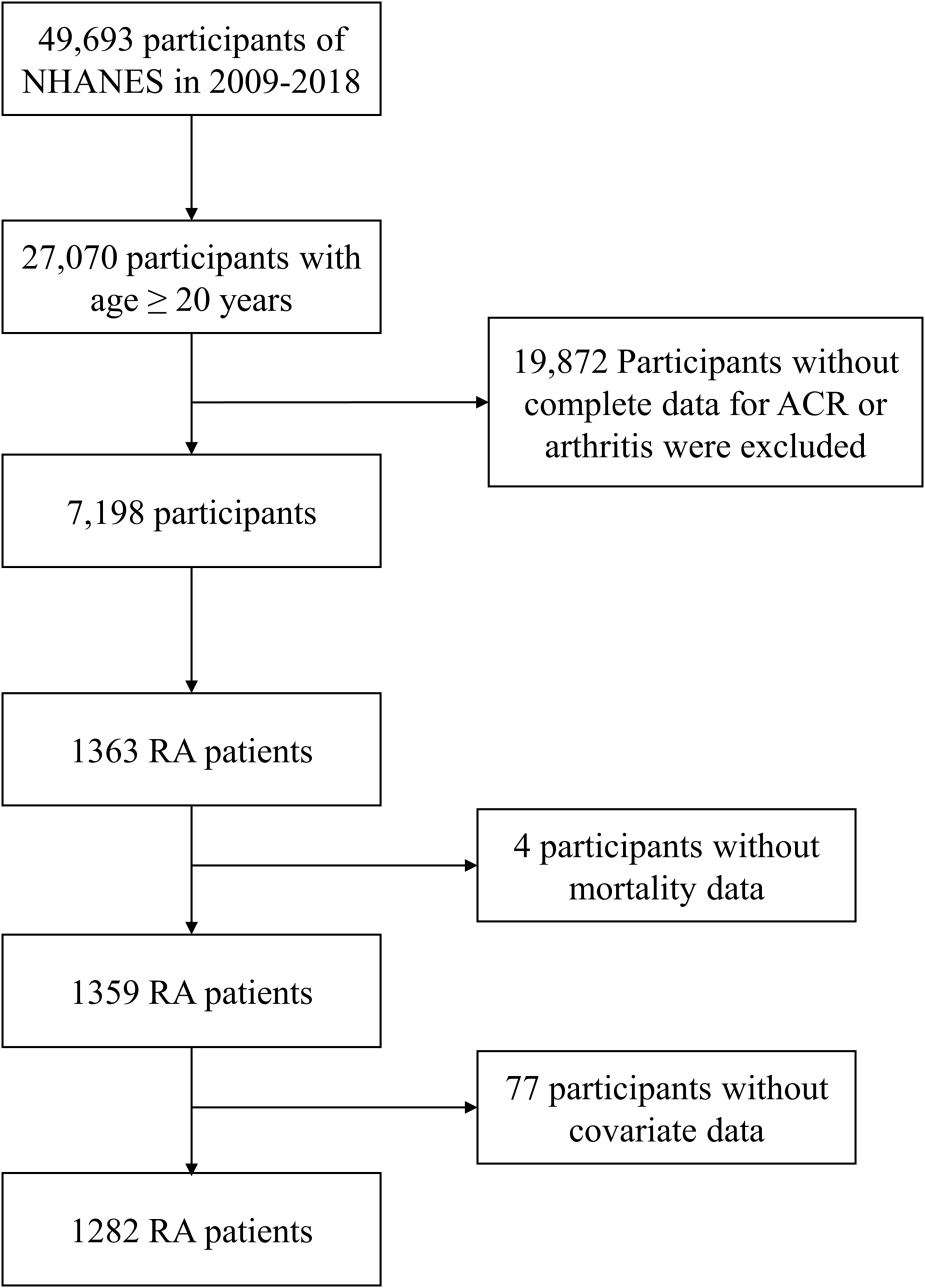
Flow chart.

A total of 1282 adult RA patients were enrolled in this study (**Figure 1**). Overall, females make up 59.05% of the population, more than males do and elderly people account for a larger percentage at 58.50%. The participants were categorized into three groups according to ACR: ACR<30 mg/g, 30-300 mg/g, and ≥300 mg/g (**Table 1**). There were 1024 RA patients with ACR less than 30 mg/g, 212 between 30 and 300, and 46 more than 300 mg/g in study population. RA cohort had proportionally more age ≥ 60 years old patients (*P <*0.001) and more patients with education level below high school as ACR increased (*P* =0.003). Besides, there were positive correlation between prevalence of diabetes, hypertension in RA participants with ACR > 300 mg/g. As ACR rises, proportion of RA participants with eGFR <60 ml/min/1.73 m2 and all-cause mortality increase. Other covariates were not significantly different among the three ACR groups.

**Table 1:** Baseline variables according to the ACR group.

characteristics	total	ACR (mg/g)			
		<30	30-300	>=300	*P*-value
	1282	1024	212	46	
Gender					0.423
male	525	418 (40.82)	84 (39.62)	23 (50.00)	
female	757	606 (59.18)	128 (60.38)	23 (50.00)	
Age					<0.001
<60	532	469 (45.80)	55 (25.94)	8 (17.39)	
>=60	750	555 (54.20)	157 (74.06)	38 (82.61)	
Race					0.195
Mexican American	194	142 (13.87)	42 (19.81)	10 (21.74)	
Non-Hispanic Black	367	293 (28.61)	60 (28.30)	14 (30.43)	
Non-Hispanic White	486	399 (38.96)	70 (33.02)	17 (36.96)	
Other	235	190 (18.55)	40 (18.87)	5 (10.87)	
Marital status					0.187
No	576	447 (43.65)	106 (50.00)	23 (50.00)	
Yes	706	577 (56.35)	106 (50.00)	23 (50.00)	
Education					0.003
Below high school	412	306 (29.88)	87 (41.04)	19 (41.30)	
High School or above	870	718 (70.12)	125 (58.96)	27 (58.70)	
PIR					0.554
Not poor	948	754 (73.63)	162 (76.42)	32 (69.57)	
poor	334	237 (23.14)	50 (23.58)	14 (30.43)	
Smoke					0.522
No	993	787 (76.86)	168 (79.25)	38 (82.61)	
Yes	289	237 (23.14)	44 (20.75)	8 (17.39)	
Diabetes					<0.001
No	859	748 (73.05)	92 (56.60)	19 (41.30)	
Yes	423	276 (26.95)	120 (43.40)	27 (58.70)	
Hypertension					<0.001
No	418	385 (37.60)	29 (13.68)	4 (8.70)	
Yes	864	639 (62.40)	183 (86.32)	42 (91.30)	
Hyperlipoidemia					0.552
No	246	191 (18.65)	44 (20.75)	11 (23.91)	
Yes	1036	833 (81.35)	168 (79.25)	35 (76.09)	
eGFR (ml/min/1.73 m2)					<0.001
<60	192	106 (10.35)	61 (28.77)	25 (54.35)	
≥60	1090	918 (89.65)	151 (71.23)	21 (45.65)	
All causes of mortality					<0.001
Heart Diseases	29	15 (1.46)	8 (3.77)	6 (13.04)	
Diabetes	10	3 (0.29)	5 (2.36)	2 (4.35)	
Pneumonia and chronic lower respiratory diseases	14	9 (0.88)	3 (1.42)	2 (4.35)	
Cerebrovascular diseases and Alzheimer’s disease	8	6 (0.59)	2 (0.94)	0 (0.00)	
Nephritis, nephrotic syndrome and nephrosis	1	0 (0.00)	0 (0.00)	1 (2.17)	
Tumor	40	31 (3.03)	6 (2.83)	3 (6.52)	
Other causes	77	50 (4.88)	22 (10.38)	5 (10.87)	
Survival	1103	910 (88.87)	166 (78.30)	27 (58.70)	

Data are shown as n% according to ACR distribution.

PIR, poverty-to-income ratio; ACR, albumin to creatinine ratio; eGFR: estimated glomerular filtration rate.

### 3.2 Association between ACR and mortality among RA patients

**Figure 2:**
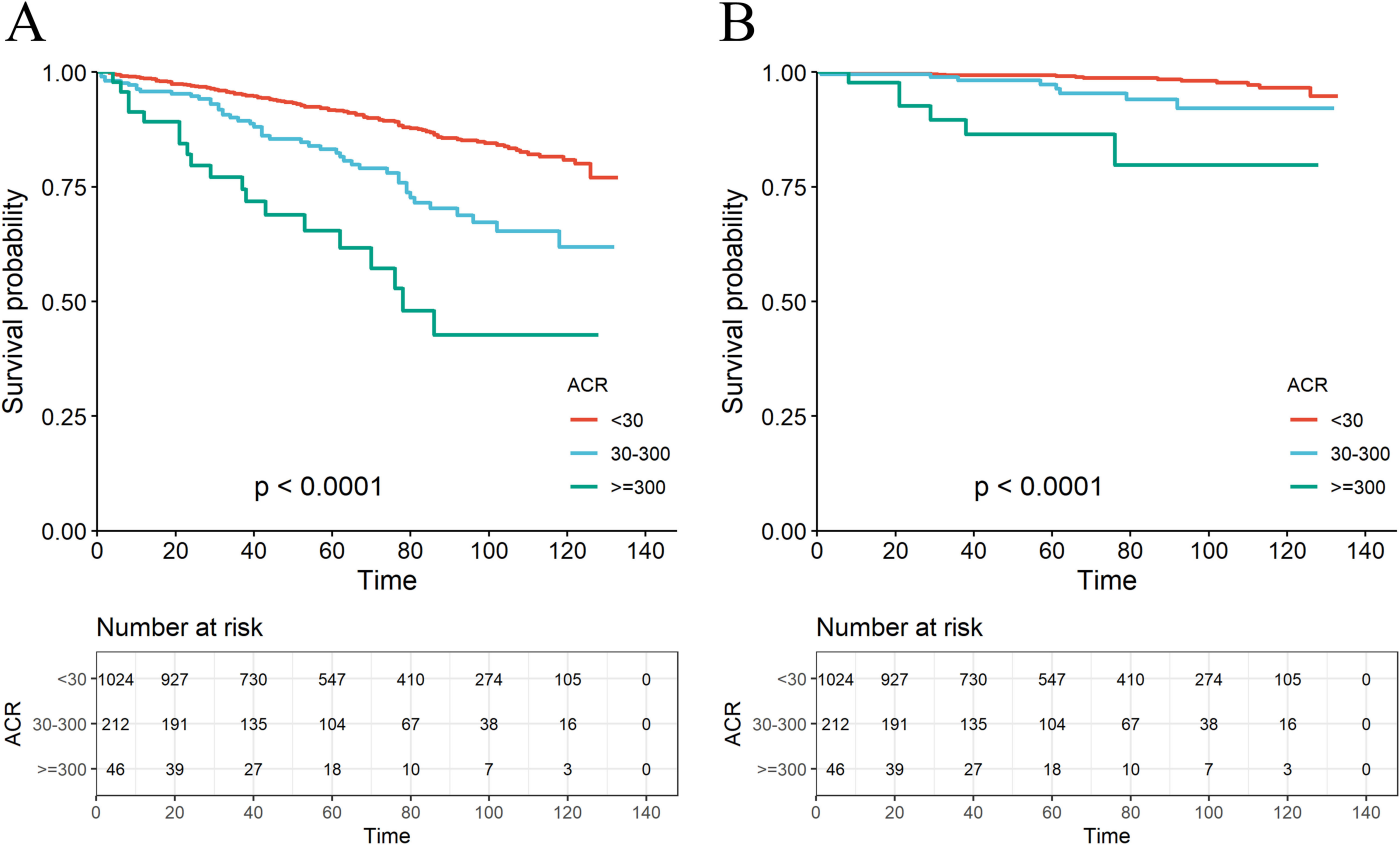
The K-M survival curve for all-cause mortality **(A)** and CVD mortality **(B)** based on the ACR group.

A total of 179 among 1282 RA patients died, with 29 fatalities attributed to cardiovascular events (**Table 2**). K-M curves based on the three ACR groups showed that the cumulative probability of survival was reduced in RA patients with high ACR compared to those with normal ACR in all groups (**Figure 2**). We observed that high ACR (30-300mg/g and ≥300mg/g groups) was associated with increased all-cause mortality compared with RA patients with normal ACR, the corresponding all-cause mortality HRs were 2.23 (95% CI 1.58,3.14) and 4.88 (95% CI 3.00,7.94) respectively (*P <*0.001). After adjusting demographic factors, RA participants with high ACR (30-300mg/g and ≥300mg/g groups) also tended to have higher odds of all-cause mortality in model 2. In model 3, the all-cause mortality of participants in the high ACR groups was also associated with higher all-cause mortality, and the HRs were 1.53 (95%CI 1.06,2.21) and 2.62 (95%CI 1.55,4.45), respectively after adjusted potential confounders. A similar but more significant trend was indicated by model 3 in the association of ACR above 300 mg/g with CVD mortality. We noticed that there was no statistical significance in the increase of CVD mortality in the overall population based on model 3, which might attribute to no apparent increase in CVD mortality of RA patients with microalbuminuria. And in other models, we could demonstrate a significant trend on mortality based on P for trend. These results suggested that ACR was a high-risk factor affecting the prognosis of RA patients, especially when ACR exceeds 300. As clinically significant proteinuria occurred, the risk of all-cause mortality and CVD mortality in RA patients significantly increased.

**Table 2:** HR and 95% CI for all-cause and CVD mortality in RA patients according to ACR groups.

	ACR			*P* for trend
	<30	30-300	>=300	
All-cause mortality				
Number of deaths (%)	114(11.13%)	46(21.70%)	19(41.30%)	
Model 1: HR (95%CI),	Ref	2.23(1.58,3.14), <0.001	4.88(3.00,7.94), <0.001	<0.001
*P*-value				
Model 2: HR (95%CI),	Ref	1.97(1.39,2.79), <0.001	3.62(2.21,5.95), <0.001	<0.001
*P*-value				
Model 3: HR (95%CI),	Ref	1.53(1.06,2.21), 0.021	2.62(1.55,4.45), <0.001	<0.001
*P*-value				
Cardiovascular mortality				
Number of deaths (%)	15(1.46%)	8(3.77%)	6(13.04%)	
Model 1: HR (95%CI),	Ref	3.09(1.31,7.31), 0.010	11.58(4.48,29.93), <0.001	<0.001
*P*-value				
Model 2: HR (95%CI),	Ref	2.25(0.93,5.43), 0.072	8.92(3.34,23.81), <0.001	<0.001
*P*-value				
Model 3: HR (95%CI),	Ref	1.36(0.56,3.34), 0.499	5.67(1.96,16.39), 0.001	0.088
*P*-value				

HR, hazard ratio; 95% CI, 95% confidence intervals; CVD, cardiovascular disease.

### 3.3 Association of ACR with mortality among RA patients with microalbuminuria

**Figure 3:**
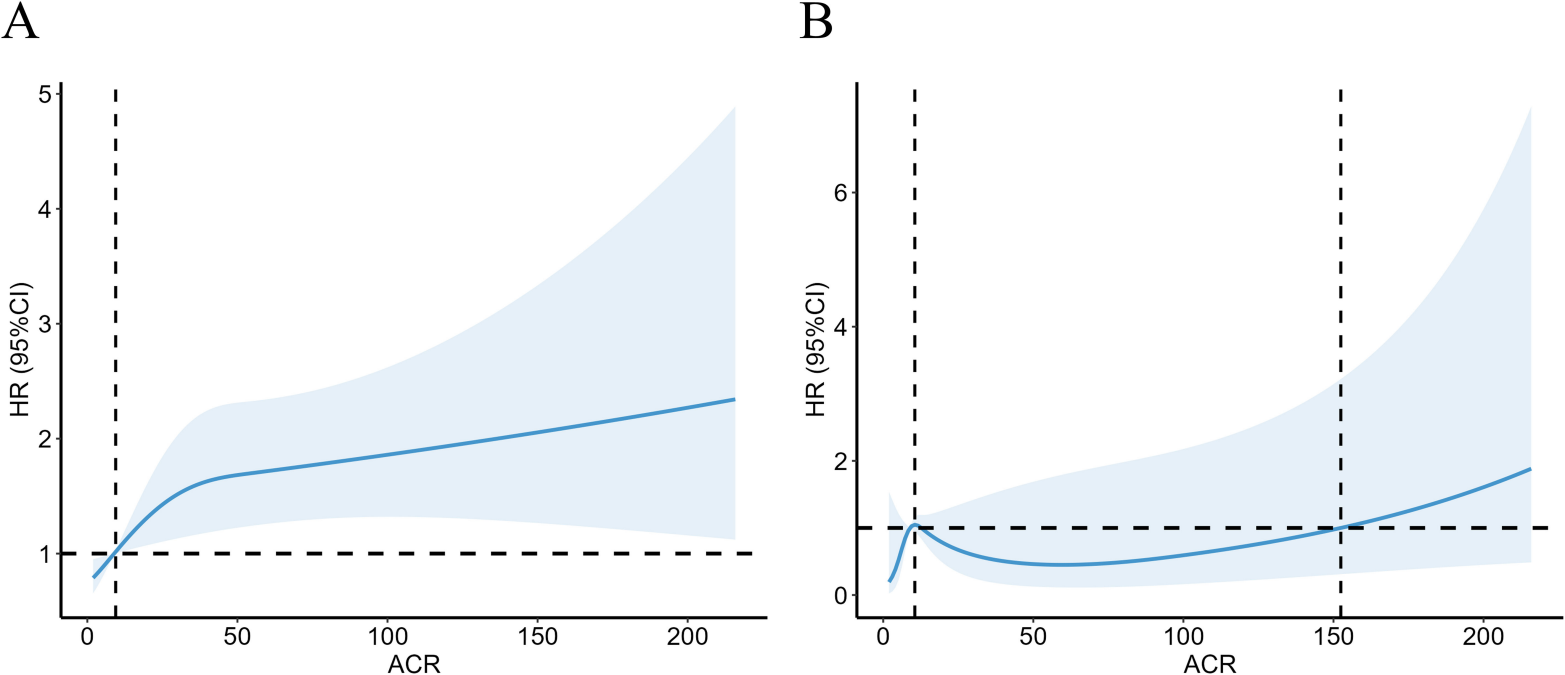
Association with all-cause **(A)** and CVD mortality **(B)** in RA at ACR <300mg/g. HR, hazard ratio; 95% CI, 95% confidence intervals; ACR, Albumin to creatinine ratio.

ACR reflects the 24-hour urinary albumin level directly as a measure of urinary microalbumin. ACR between 30-300 mg/g indicates elevated urinary microalbumin, however, model 3 above suggested there was no significant correlation between microalbumin excretion and CVD mortality for RA. We further investigate the non-linear relationship between mortality and ACR at this interval. RCS revealed a nonlinear correlation for all-cause mortality in RA patients with microalbuminuria (**Figure 3A**, *P* =0.0014, *P* for nonlinear=0.0329). For RA patients with microalbuminuria, there was no correlation between ACR and their CVD mortality (**Figure 3B**, *P* =0.173, *P* for nonlinear=0.204). However, RCS still showed an upward trend of CVD mortality as ACR exceeded 152.44 mg/g.

### 3.4 Subgroup analysis of all-cause and CVD among RA patients with microalbuminuria

**Figure 4:**
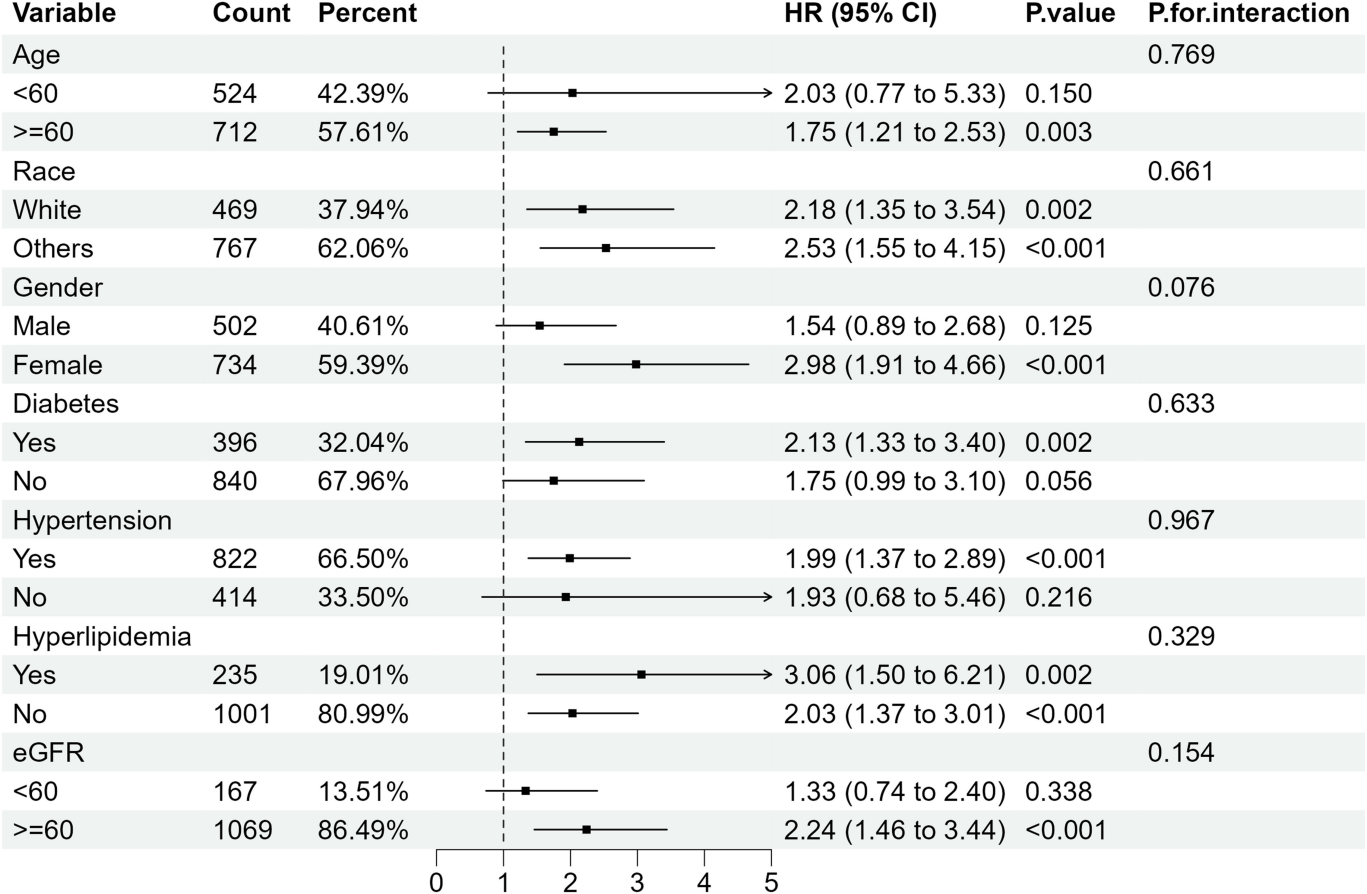
Subgroup analysis of all-cause mortality in RA patients with ACR < 300 mg/g. HR, hazard ratio; 95% CI, 95% confidence intervals; ACR, Albumin to creatinine ratio.

**Figure 5:**
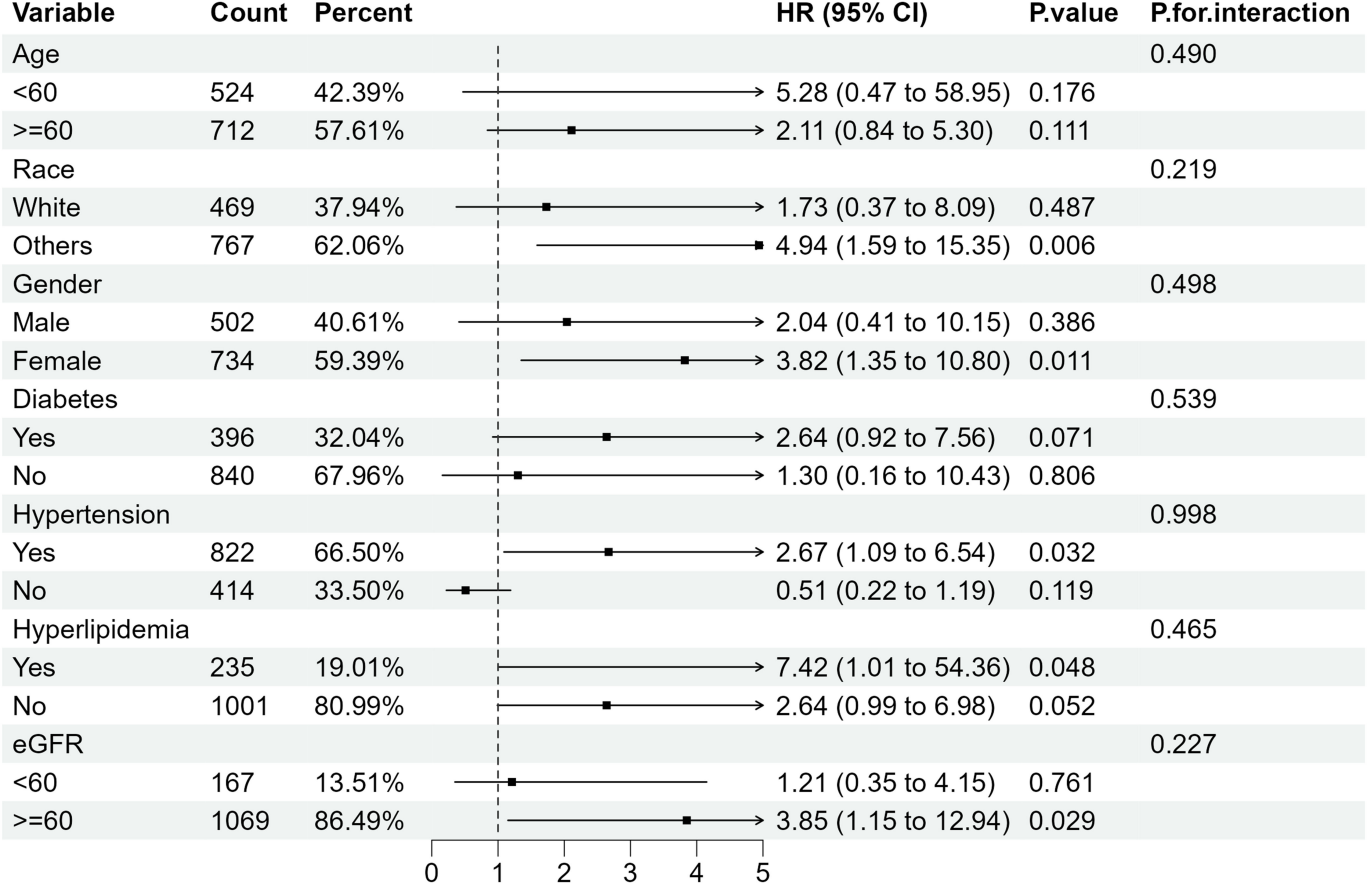
Subgroup analysis of CVD mortality in RA patients with ACR < 300 mg/g. HR, hazard ratio; 95% CI, 95% confidence intervals; ACR, Albumin to creatinine ratio.

Further subgroup analysis of RA individuals with ACR <300 mg/g stratified by sex, age, ethnicity, diabetes mellitus, hypertension, hyperlipidemia and eGFR showed that when microalbuminuria existed, the all-cause mortality rate was higher in RA patients with the following characteristics: older, female, eGFR≥60 ml/min/1.73 m2 and those with hypertension or diabetes. Despite the existence of hyperlipidemia, all-cause mortality increased in RA patients with albuminuria, of which the HRs were 3.06(95%CI 1.50, 6.21) and 2.03 (95%CI 1.37, 3.01) compared with RA patients with normal ACR (**Figure 4**). Regarding the effect of ACR on CVD mortality in RA patients, subgroup analyses suggested that CVD mortality was higher in participants with the following characteristics: female, other ethnicity, eGFR≥60 ml/min/1.73 m2, those with hypertension or hyperlipidemia (**Figure 5**). In conclusion, female RA participants with microalbuminuria who concurrently suffer from hypertension or hyperlipidemia are at higher risk of having their prognosis significantly affected by ACR, even though their eGFR levels are over 60 ml/min/1.73 m2.

### 3.5 Sensitivity analysis

The propensity score of patients with ACR less than 300 mg/g was calculated and matched. After propensity score matching (PSM), all-cause mortality for RA patients with microalbuminuria was 1.72 times (95%CI 1.08,2.76) higher than that of RA patients with ACR less than 30 mg/g (**Table 3**), suggesting that all-cause mortality was still associated with ACR; Whereas, its elevated CVD mortality was not statistically significant, consistent with the previous results.

**Table 3:** Association of all-cause mortality and CVD mortality with ACR in RA patients with ACR < 300 mg/g after PSM.

	ACR		*P-*value
	<30	30-300	
All-cause mortality			
Model 1: HR (95%CI)	Ref	1.70(1.08,2.70)	0.023
Model 2: HR (95%CI)	Ref	1.64(1.03,2.60)	0.035
Model 3: HR (95%CI)	Ref	1.72(1.08,2.76)	0.022
Cardiovascular mortality			
Model 1: HR (95%CI)	Ref	2.25(0.68,7.46)	0.187
Model 2: HR (95%CI)	Ref	2.25(0.67,7.50)	0.187
Model 3: HR (95%CI)	Ref	2.76(0.81,9.37)	0.105

HR, hazard ratio; 95% CI, 95% confidence intervals; ACR, Albumin to creatinine ratio.

### 3.6 The prognostic efficacy of ACR and eGFR for all-cause mortality and CVD mortality

**Figure 6:**
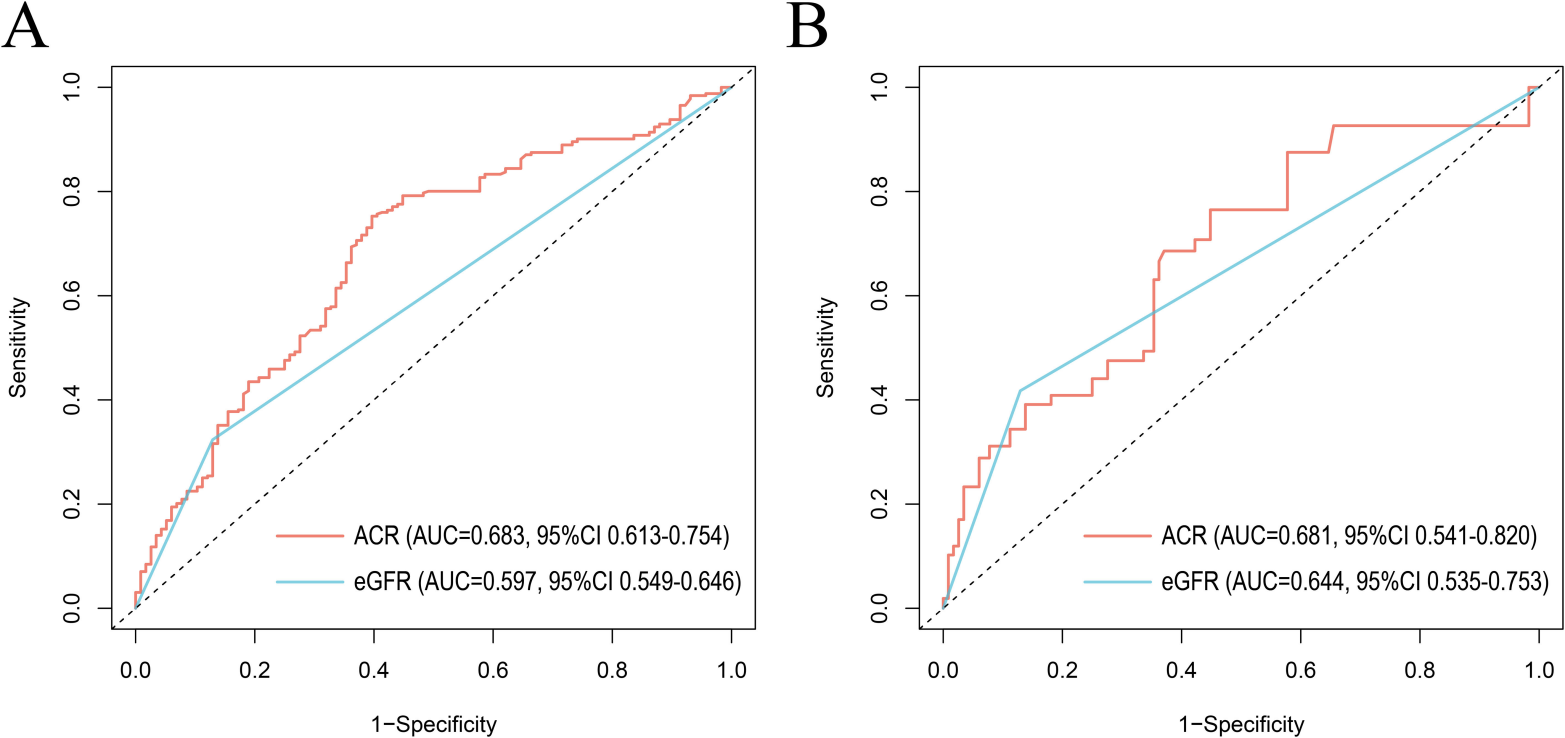
ROC curves of ACR and eGFR for all-cause mortality **(A)** and CVD mortality **(B)** at 10 years. ROC, The receiver operating characteristic; ACR, albumin to creatinine ratio; eGFR, estimated glomerular filtration rate. AUC, area under the curve; 95% CI, 95% confidence intervals.

The receiver operating characteristic (ROC) curves were utilized to evaluate the prognostic value of ACR and eGFR for all-cause mortality (**Figure 6A**) and CVD mortality (**Figure 6B**) in RA patients. Compared with eGFR, ACR offered better prognostic efficacy than eGFR with higher AUC values in ten years. The AUC values of ACR for all-cause mortality and CVD mortality were 0.683 (95% CI 0.613-0.754) and 0.681 (95% CI 0.541-0.820) respectively, followed by eGFR (AUC for all-cause mortality: 0.597, 95% CI 0.549-0.646 and AUC for CVD mortality: 0.644, 95% CI 0.535-0.753).

## 4 Discussion

Our study showed that the urinary albumin to urinary creatinine ratio was an independent risk factor for all-cause mortality in RA patients, stratified by age, race, gender, eGFR, and other comorbidities. In RA patients with modestly elevated albuminuria (30-300 mg/g), there was a 53% increase in all-cause death compared to RA patients with normal urine albumin excretion. As is shown in RCS plot (**Figure 3A**), there was a steady trend toward higher mortality in the RA population with microalbuminuria. When urinary albumin excretion was markedly elevated (≥300 mg/g), all-cause mortality of RA patients was 2.62 times higher than in those with normal ACR (<30 mg/g). ACR is an essential threat contributing to cardiovascular events in normal populations (13, 14). However, in our study, when all variables were taken into account, the RA patients did not exhibit a statistically significant increase in cardiovascular mortality as urine albumin excretion modestly raised (30-300 mg/g). Based on subgroup analysis, we further discovered that a mild increase of CVD mortality was seen in RA patients who were female, of other races, eGFR above 60 ml/min/1.73 m2 and had hypertension or hyperlipidemia. Noticeably, patients with considerably higher urine protein excretion (≥300 mg/g) may have a 4.67-fold increase in cardiac cause mortality compared to those with normal ACR.

The common pathological types of rheumatoid arthritis patients combined with renal dysfunction remained controversial. It was believed that common types of RA renal biopsies included mesangial proliferative glomerulonephritis, IgA nephropathy and membranous glomerulonephritis (15, 16). One of important clinical manifestations of these complications is increased urinary protein excretion. During the early stage of renal involvement in rheumatoid arthritis, no clinical abnormally levels are detected on creatinine, urea nitrogen, and 24-hour urinary albumin. For the purpose of evaluating subclinical renal impairment in rheumatoid arthritis, ACR is currently essential due to its sensitivity as a renal pathological test. Therefore, in our analysis we focused primarily on the predictive significance of microalbuminuria (ACR < 300 mg/g) in individuals with RA. Clinical research on microalbuminuria in RA has demonstrated that RA disease duration is closely correlated with ACR, which is significantly greater in RA patients than in normal individuals (17). The causes of renal insufficiency in patients with RA are still up for debate. These include vasculitis, amyloidosis, or renal injury caused by pharmacological agents. Increased vascular permeability in the inflammatory environment of patients with RA may lead to alter glomerular vasculature penetration to plasma albumin, which consequently raises urinary albumin excretion. Renal damage caused by amyloidosis typically has a long latency period and is asymptomatic in the early stages before producing significant quantities of proteinuria (18). Additionally, pharmacological factors are the most common reported causes of renal insufficiency in RA A prospective study showed that NSAIDs hastened the deterioration of renal function in RA patients with advanced kidney damage as some DMARDs did (19). Concomitant use of methotrexate and NSAIDs increased risk of acute renal failure significantly. And methotrexate monotherapy may exacerbate renal insufficiency in RA patients with renal dysfunction (20, 21). Our study demonstrated that elevated ACR significantly increased the risk of mortality in RA patients regardless of micro- or macro-proteinuria; Therefore, early identification of renal injury in RA patients by ACR facilitates physicians to clarify development of diseases and promptly adjust potentially nephrotoxic drugs to improve the prognosis of RA patients.

In addition to being an important indicator for assessing renal insufficiency, ACR can predict the occurrence of cardiovascular events in people with RA to some extent. It has been found that elevated urinary albumin excretion increases the prevalence of cardiovascular complications such as atherosclerosis in patients with RA (22–24). Due to the chronically elevated inflammatory burden in RA patients, they have a higher CVD risk than the normal group. Urinary albumin excretion can be regarded as an important indicator for assessing CVD mortality of RA populations, for reasons that may include either: 1) It reflects endothelial dysfunction or 2) It may indicate the acute phase inflammatory response. Urinary albumin excretion mirrors serum albumin levels which serve an important role in maintaining endothelial integrity. Serum albumin lessens endothelial dysfunction by directly inhibiting oxidative stress and inflammatory pathways (25). It also carries substances that scavenge free radicals, like sphingosine-1-phosphate, which shields the endothelium (26). Besides, an association has been found between urinary protein excretion and vascular Willebrand factor (vWF), a hemostatic factor released in response to endothelial cell damage. Both ACR and vWF could embody endothelial damage and increased permeability, contributing to atherosclerotic plaque formation and atherosclerosis (27). Urinary albumin excretion varies with inflammatory cytokine secretion which further promotes plaque rupture during the acute inflammatory phase. An early myocardial infarction is characterized by a brief and transient rise in urine albumin, especially microalbuminuria, without functional or structural renal damage. A cox proportional risk model has revealed that urinary albumin excretion rate was a better predictor of in-hospital mortality than Killip class or left ventricular ejection fraction (28).

We found that although an increase in cardiovascular mortality was observed by restricted cubic spline plots when ACR exceeded 152.44 mg/g, there was no statistically significant increase in cardiac cause-specific mortality in the presence of microalbuminuria in RA patients compared with those with normal urinary albumin excretion. This suggests that in contrast to the general population, microalbuminuria in RA patients has little effect on CVD mortality within a certain range. We speculate that this may be due to the fact that renal disease was the primary cause of microalbumin excretion in RA patients, with a relatively weak correlation to cardiovascular events. However, the impact of ACR on CVD mortality increased dramatically over 300 mg/g, indicating that ACR may be an effective predictor of an adverse prognosis for cardiovascular events in people with RA to some extent. To access its prognostic efficacy, ROC curves were plotted and suggested that ACR hold a higher prognostic value for both all-cause mortality and CVD mortality in RA compared to eGFR, another indicator usually used for CKD assessment. Meanwhile, ACR testing is an affordable and non-invasive tool in clinical practice.

Microalbuminuria measurement has not been popularized in the clinic for patients with rheumatic diseases, despite the fact that ACR has been extensively utilized in the assessment and diagnosis of CKD (29, 30). The above advantages may allow us to choose ACR as one of the routine tests for RA patients. It helps early recognition of adverse prognosis especially for targeted populations. Our subgroup analysis suggested that RA patients characterized by the following features: female, other ethnicity, eGFR≥60 ml/min/1.73 m2, hypertension or hyperlipidemia share a higher CVD risk and seem to be targeted testing population. Other researchers found that patients with RA disease of more than 10 years, positive RF, positive ACPA and presence of extra articular manifestations are at a higher risk for CVD (31). ACR testing may be more necessary under those conditions. Based on the NHANES, this study covered over 1,000 RA patients and assessed the independent impact of urinary albumin excretion on the prognosis of adult RA patients in the United States. Our study not only revealed that the association between ACR and mortality in RA participants, also proposed that ACR can be considered as a regular marker for renal assessment and outcome prediction in RA. However, the current analysis still has some limitations. Firstly, RA data collected from NHANES belongs to questionnaire data, with a definition based on “What type of arthritis does it belong to”. It may result in recall bias. Secondly, NHANES is still lacking for more refined statistics about RA specific antibodies such as anti-citrullinated protein antibody as a public database. Besides, a small amount of data on CVD mortality in the cohort may affect statistical analysis of association of ACR with adverse outcomes. Therefore, large prospective cohort studies based on more detailed clinical characteristics are still essential for further validating the correlation between ACR and the prognosis of RA patients.

## 5 Conclusion

After adjustment for relevant covariates including demographic, lifestyle and comorbidity factors, ACR was an important independent risk factor affecting the prognosis of adult RA patients. There was an increase in all-cause mortality in the groups with microalbuminuria or macroalbuminuria. In our investigation, ACR did not significantly correlate with cardiovascular death in RA patients with microalbuminuria. However, ACR did significantly correlate with cardiovascular death in cases of macroalbuminuria. Overall, ACR was closely related to the prognosis of RA patients and could be considered as a sensitive and independent indicator for physicians to predict the mortality especially CVD mortality in the process of diagnosis and treatment.

## Data Availability

Publicly available datasets were analyzed in this study. This data can be found here: The data underlying this article are available in NHANES (https://www.cdc.gov/nchs/nhanes/index.htm).
